# Nanospherical like reduced graphene oxide decorated TiO_2_ nanoparticles: an advanced catalyst for the hydrogen evolution reaction

**DOI:** 10.1038/srep20335

**Published:** 2016-02-01

**Authors:** Dejian Chen, Liling Zou, Shunxing Li, Fengying Zheng

**Affiliations:** 1 College of Chemistry and Environment, Minnan Normal University, Zhangzhou, 363000, China

## Abstract

Modification of titanium dioxide (TiO_2_) for H_2_ generation is a grand challenge due to its high chemical inertness, large bandgap, narrow light-response range and rapid recombination of electrons and holes. Herein, we report a simple process to prepare nanospherical like reduced graphene oxide (NS-rGO) decorated TiO_2_ nanoparticles (NS-rGO/TiO_2_) as photocatalysts. This modified TiO_2_ sample exhibits remarkably significant improvement on visible light absorption, narrow band gap and efficient charge collection and separation. The photocatalytic H_2_ production rate of NS-rGO/TiO_2_ is high as 13996 μmol g^−1^ h^−1^, which exceeds that obtained on TiO_2_ alone and TiO_2_ with parallel graphene sheets by 3.45 and 3.05 times, respectively. This improvement is due to the presence of NS-rGO as an electron collector and transporter. The geometry of NS-rGO should be effective in the design of a graphene/TiO_2_ composite for photocatalytic applications.

The production of H_2_ by solar energy conversion has been considered as one of the major strategies for solving the global energy problem^1^. Since the first report by Fujishima and Honda^2^ on photoelectrochemical water splitting on a TiO_2_ electrode, the photocatalytic H_2_ production has attracted a lot of attention. Various semiconductor photocatalysts, such as TiO_2_^3^,^4^, ZnO^5^,^6^, CdS^7^,^8^,^9^, C_3_N_4_^10^,^11^,^12^, WO_3_^13^, and BiVO_4_^14^ have been investigated. Most of them suffer from wide band gap, photocorrosion, and low separation efficiency of electron-hole pairs^3^,^4^,^5^,^6^,^7^,^8^,^9^. Many efforts including doping, composites, noble metal loading, heterojunction fabrication and sensitization by dyes or quantum dots have been made to overcome these problems^15^,^16^. Among these, constructing nanocomposites is a promising approach to obtain high performance photocatalysts.

Graphene, a two-dimensional layer of sp^2^-hybridized carbon atoms, has been widely used in sensors, electronics, drug delivery, supercapacitors and catalysis due to its unique electrical properties^17^,^18^, high thermal conductivity^19^, mechanical strength and specific surface area^20^. To date, a variety of reduced graphene oxide (rGO) based semiconductor photocatalysts (e.g., TiO_2_, CdS, BiVO_4_ and C_3_N_4_) have been reported^21^,^22^,^23^,^24^,^25^,^26^,^27^. Nanocomposite is currently one of the most interesting subjects for photocatalytic, especially graphene/nano-TiO_2_ composites^28^,^29^,^30^,^31^,^32^,^33^,^34^,^35^,^36^,^37^,^38^. Although the role of rGO for photocatalytic performance has been extensively investigated, the complexity of the rGO structure, including hybridized atoms, various functional groups, and defects makes it difficult to well understand some electrical and optical properties, and thus some new explorations in developing highly efficient photocatalysts have been hampered. The introduction of graphene can enhance photocatalytic the performance because both specific reaction sites and photoresponding range are improved^7^. This work confirm that the pathway of electrons collection and separation may also be a key factor in the photocatalytic performance but until now it has completely been ignored. The material properties are strongly dependent on their size, shape and, equally important, structures^39^. Nanostructure with controllable morphology not only facilitate mass transfer in catalysis, but more importantly can assist to guide charge movement to accelerate the collection and separation of electron-hole pairs at materials interface for efficient utilization of solar energy to drive relevant reactions^10^. As far as we are aware, few attempt has been made to synthesize nanospherical reduced graphene oxide (NS-rGO), and hence, its unique photocatalytic activity is almost completely unknown^40^,^41^,^42^,^43^. Herein, we synthesized the NS-rGO decorated TiO_2_ nanoparticle through the core shell method followed by mixing. This method is interesting for the preparation of NS-rGO decorated TiO_2_ nanoparticle. The photocatalytic H_2_ production rate of NS-rGO/TiO_2_ is enhanced than that obtained on TiO_2_ alone and TiO_2_ with parallel graphene sheets by 3.45 and 3.05 times, respectively.

## Results

**Figure 1:**
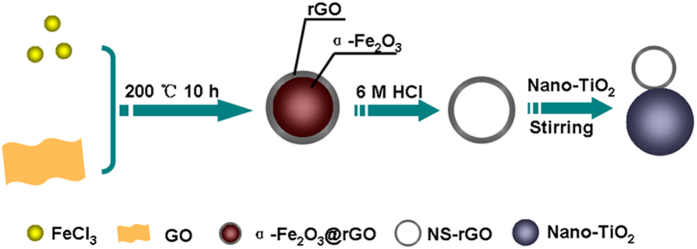
Illustration of NS-rGO/TiO_2_ synthesis.

In line with the intensive research on layer graphene and carbon nanosphericals, we report here for the first time an approach for the facile preparation of NS-rGO decorated TiO_2_ heterojunction materials. The morphology and structure of α-Fe_2_O_3_@rGO were characterized by SEM and TEM. As illustrated in Fig. 1, core-shell α-Fe_2_O_3_@rGO (Figures S1 and S3) with monodisperse and well-defined graphene shell^40^ is an ideal template to prepare NS-rGO. According to the result in Fig. S1b, the structure of the prepared α-Fe_2_O_3_@rGO was between sphere and ellipse. The Figures S2a,b respectively showed the SEM and TEM images of α-Fe_2_O_3_@rGO, which further supporting our judgment. After the removal of α-Fe_2_O_3_ by HCl, spherical like NS-rGO with lofted surface was observed in Figure S4, because NS-rGO was prepared by acidic etching. The nanosized NS-rGO with spherical like morphology is used for decorating TiO_2_ nanoparticles. The strong interfacial interaction between positively charged NS-rGO and negatively charged TiO_2_ nanoparticals can extend photoresponding range and suppress photogenerated carriers’ recombination. The NS-rGO decorated TiO_2_ nanocomposites exhibit much higher photocatalytic activities for water splitting into H_2_ than layer rGO decorated TiO_2_ nanocomposites. This work may provide new insights into the fabrication of graphene based nanocomposite photocatalysts for various applications.

**Figure 2:**
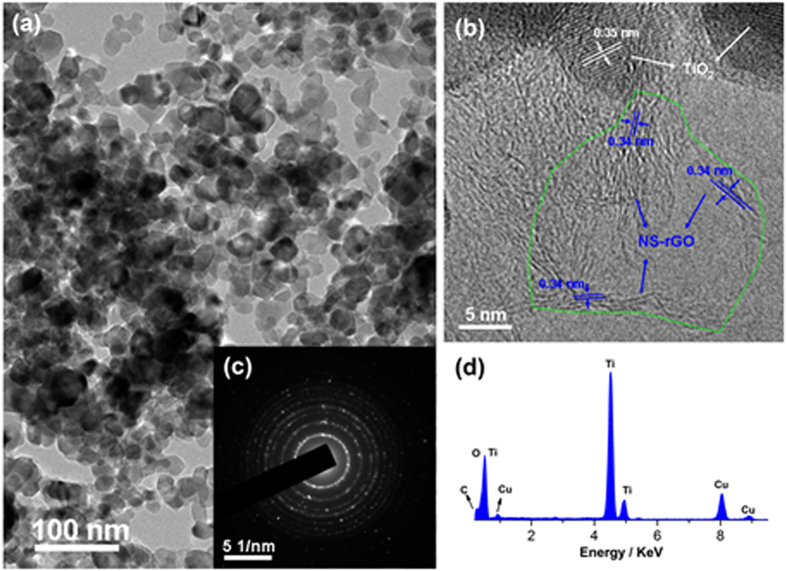
Typical TEM (**a**) and HRTEM (**b**) images of NS-rGO/TiO_2_ nanocompositions, the corresponding SAED pattern (**c**) and EDX pattern (**d**).

In a typical experiment, with a mass ratio of 100:1, TiO_2_ and NS-rGO were mixed at room temperature (about 20 °C), resulting in a gray suspension. TEM was employed to observe its morphology. Figure 2a clearly showed that these nanocomposites were sphere structure with nanosize, and no large reduced graphene oxide layer could be seen. Figure 2b taken from the interfacial region indicated that NS-rGO was loaded onto TiO_2_ nanoparticles and the interplanar distance of TiO_2_ and NS-rGO in the crystalline network was 0.35 nm and 0.34 nm, respectively. Selected-area electron diffraction (SAED) pattern taken from nanocomposites showed the multi-crystalline nature of nanoparticles (Fig. 2c). In addition, energy dispersive X-ray spectrum (EDX) of the NS-rGO/TiO_2_ nanocomposites (Fig. 2d) showed the existence of three elements, Ti, O, and C. For rGO/TiO_2_ nanocomposites, Figure S5a clearly showed that the nanocomposites of rGO sheet and TiO_2_ nanoparticles, the sheet structure of rGO was observed in blue line area, indicated forming composite structure among them. Figure S5b clearly showed that the Pt nanoparticles were uniform deposited on the surface of NS-rGO/TiO_2_ nanocompositions.

**Figure 3:**
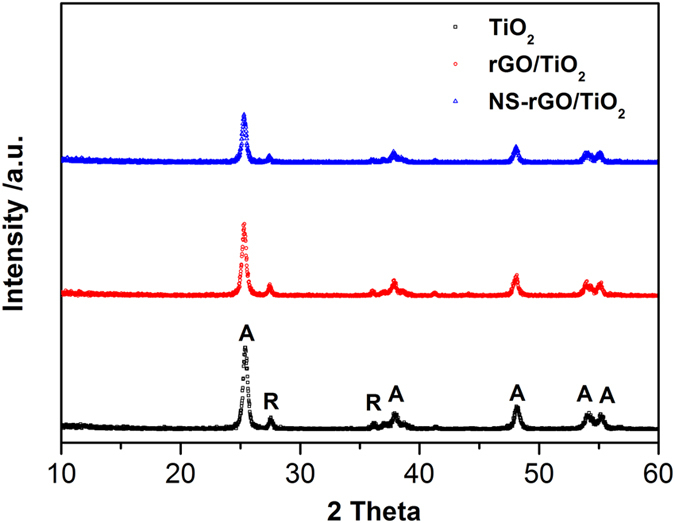
XRD patterns for as-prepared TiO_2_, rGO/TiO_2_, and NS-rGO/TiO_2_.

XRD pattern (Fig. 3) was recorded for bare TiO_2_, rGO/TiO_2_, or NS-rGO/TiO_2_ to confirm and investigate the influence of introduction materials on the crystallinity of TiO_2_ nanoparticles. rGO/TiO_2_ nanocompositions were synthesized for the comparation with NS-rGO/TiO_2_ in the subsequent section. For TiO_2_, one could see that rutile and anatase were mixed. The nanocomposites of rGO/TiO_2_, and NS-rGO/TiO_2_ with a same and low carbon content exhibited anatase and rutile phases from TiO_2_. Due to very little of graphene introduction, no peaks corresponding to the presence of graphite latticewere recorded in the pattern. The results implied that the existence of graphene materials did not change the crystal phase of TiO_2_.

**Figure 4:**
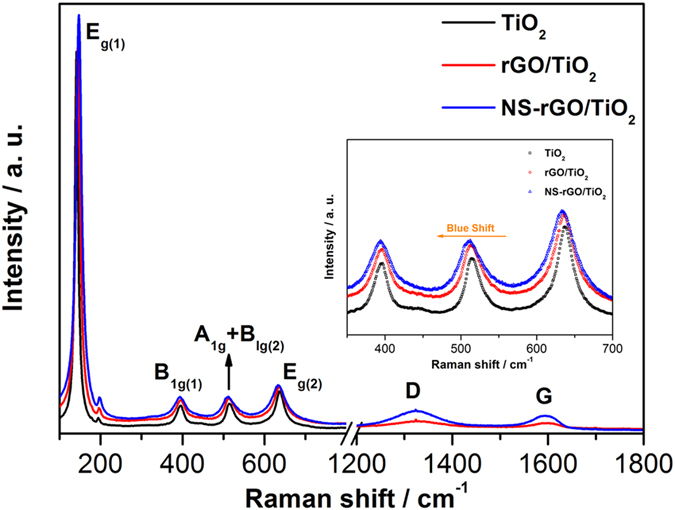
Raman spectra of TiO_2_, rGO/TiO_2_, and NS-rGO/TiO_2_ nanocompositions.

To clarify this issue further, raman analysis was performedand shown in Fig. 4. The characteristic peaks of bare TiO_2_, rGO/TiO_2_ and NS-rGO/TiO_2_ composites were observed at about 141, 396, 514, and 637 cm^−1^, corresponding to the E_g(1)_, B_1g(1)_, A_1g_ + B_1g(2)_, and E_g(2)_ modes of anatase, respectively^27^. Also, the observed B_1g(1)_, A_1g_ + B_1g(2)_, and E_g(2)_ peaks of the rGO/TiO_2_ and NS-rGO/TiO_2_ composites were slightly shifted in comparison with the bare TiO_2_, indicated the formation of hybrid structure between NS-rGO and TiO_2_ nanoparticles. Significantly, two peaks at about 1356 and 1596 cm^−1^, which were corresponded to the well-defined D and G bands for the graphitized structures, respectively, were also observed. This result confirmed the presence of reduced graphene oxide in the rGO/TiO_2_ and NS-rGO/TiO_2_ composites. The intensity ratio of the D and G bands (I_D_/I_G_) of rGO/TiO_2_ and NS-rGO/TiO_2_ nanocompositions was respectively 1.18 and 1.24, which indicated that the difference in micro-structures and NS-rGO had fewer defects than rGO. Compared to the I_D_/I_G_ of GO (0.96), the ID/IG of rGO was increased, which indicated that GO could be reduced with hydrothermal reaction. This increase might be caused by structural defects within the sp^2^ carbon network that arose upon the reduction of the exfoliated GO.

**Figure 5:**
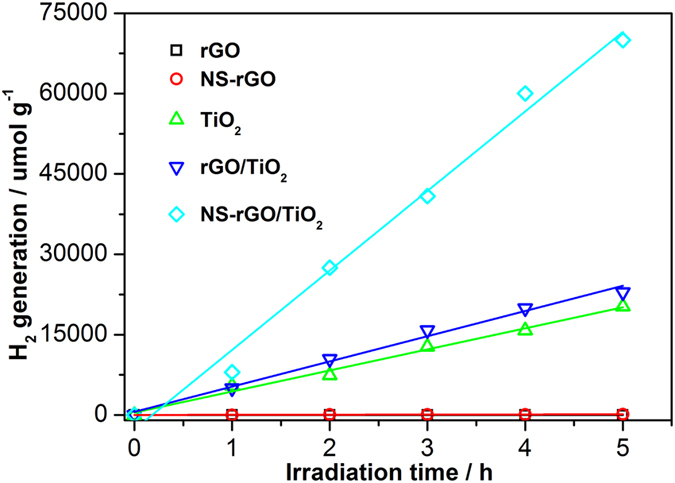
Reaction time profiles of hydrogen production under UV-Vis light illumination over TiO_2_, rGO/TiO_2_ and NS-rGO/TiO_2_ compositions for comparsion. (reaction conditions: light source, 300 W Xe arc lamp; catalyst, 50 mg; methanol aqueous solution, 20% v/v 80 mL.) All data at least 3 times reproduced measurements.

In this work, the photocatalytic H_2_ production activity of the prepared rGO, NS-rGO, TiO_2_, rGO/TiO_2_, and NS-rGO/TiO_2_ samples was evaluated under UV-Vis irradiation using methanol and Pt as sacrificial reagent and co-catalyst, respectively. As shown in Fig. 5, graphene exhibited a significant influence on the photocatalytic activity. Compared to bare TiO_2_, the enhancement of photocatalytic activity for rGO/TiO_2_ (3.05 times) and NS-rGO/TiO_2_ (3.45 times) composites was observed. For TiO_2_ alone, a relatively low photocatalytic H_2_ production rate (4053 μmol g^−1 ^h^−1^) was obtained due to its rapid recombination of conduction band electrons and valence band holes. Controlled experiments used rGO and NS-rGO as catalyst at the same conditions, graphene materials showed no significant photocatalytic activity for H_2_ production. The photocatalytic H_2_ production rate of hybrid materials were significantly greater than that of either TiO_2_ or graphene materials. The NS-rGO/TiO_2_ had a higher H_2_ production rate (13996 μmol g^−1 ^h^−1^) than rGO/TiO_2_ (4588 μmol g^−1 ^h^−1^) and TiO_2_ (4053 μmol g^−1 ^h^−1^)in the five hours. The compared the rate of hydrogen evolution with the activities obtained in similar systems in previous investigations are influenced by different experimental conditions (i. e., catalyst concentration, light intensity). Therefore, the accurate comparison between other materials is very difficult.

## Discussion

The introduction of carbon materials (e.g., fullerene^44^, carbon dots^45^, carbon nanotubes^46^, graphene oxide and graphene sheets^21^,^24^) into semiconductor could significantly enhance photocatalytic activity. The enhancement mechanism could be attributed to carbon materials, which could (1) offer more active adsorption sites and photocatalytic reaction centers, (2) suppress the recombination of the photogenerated electron/hole pairs, (3) prolong the lifetime of electrons and holes, (4) narrow the band gap of photocatalyst, and (5) act as photosensitizer or cocatalyst for catalytic reaction.

**Figure 6:**
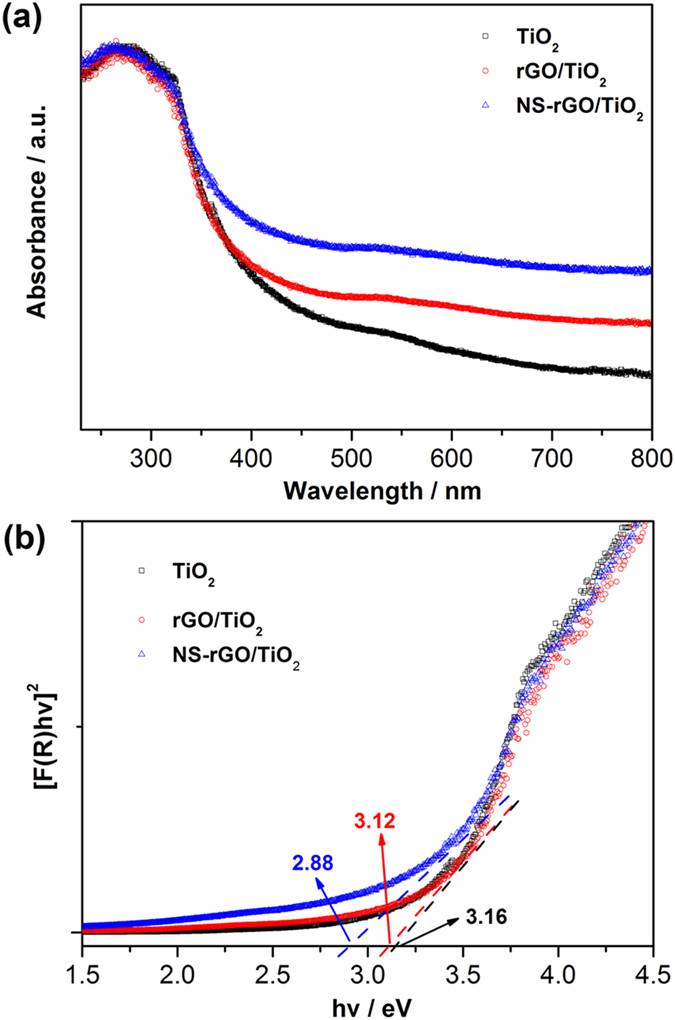
(**a**) UV-vis absorption spectra of bare TiO_2_, rGO/TiO_2_ and NS-rGO/TiO_2_. (**b**) Plot of transformed Kubelka-Munk function (*F(R)hv*)^1/2^ versus energy of light (*hv*) for bare TiO_2_, rGO/TiO_2_ and NS-rGO/TiO_2_.

UV-vis diffuse reflectance spectra (DRS) were used to probe the optical properties of the bare TiO_2_, rGO/TiO_2_ and NS-rGO/TiO_2_ composites. According to Fig. 6a, the light absorbance of rGO/TiO_2_ and NS-rGO/TiO_2_ nanocomposites was extended into the visible regime (>450 nm), which was in faithful agreement with the color of the samples (i.e., white for bare TiO_2_, french grey for rGO/TiO_2_ and dark gray for NS-rGO/TiO_2_). The red shift and the improved light absorption for rGO/TiO_2_ and NS-rGO/TiO_2_ could be attributed to the introduction of graphene, XPS and FTIR showed no Ti-O-C species in compositions (Figure S6). A plot obtained via the transformation based on Kubelka-Munk function versus energy of light was shown in Fig. 6b. The estimated bandgaps of the samples were 3.16, 3.12, and 2.88 eV, corresponding to bare TiO_2_, hybrid materials with rGO and NS-rGO, respectively. This results indicated a bandgap narrowing of TiO_2_ integrated with rGO or NS-rGO by the interfacial interaction. Because of light absorbance increase and bandgap narrowing, a more efficient utilization of light could be obtained.

**Figure 7:**
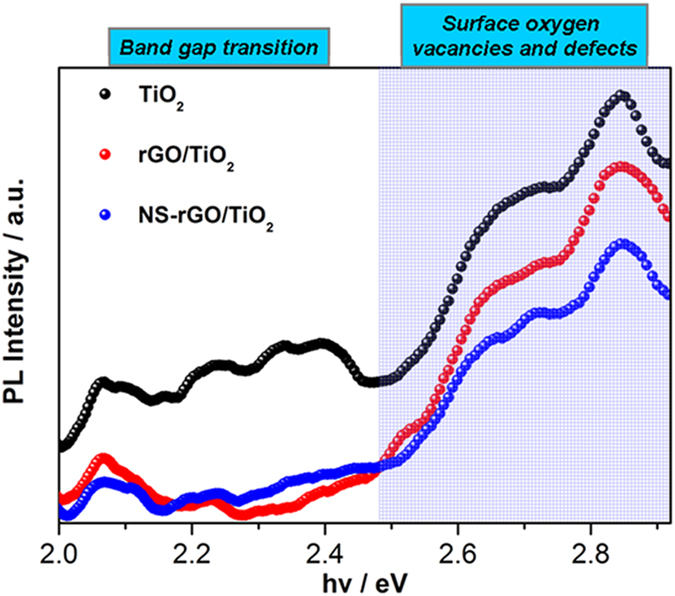
PL spectra of bare TiO_2_, rGO/TiO_2_ and NS-rGO/TiO_2_ (with the excitation wavelength of 315 nm).

To further understand the transfer and recombination processes of photoexcited charge carriers in these samples, PL spectra of bareTiO_2_, rGO/TiO_2_ and NS-rGO/TiO_2_, which could be employed to investigate the fate of electron-hole pairs in semiconductor successfully^47^,^48^, were also measured and shown in Fig. 7. As could be seen from this figure, TiO_2_, with mixture phases, was characterized by three main peaks (around 2.4, 2.72, 2.85 eV) and lots of small peaks, which attributed to band gap transition and surface oxygen vacancies and defects^44^. The composite of graphene with TiO_2_ could influence its PL spectrum. But the main emission of origin materials were not changed. Obviously, in comparison to bare TiO_2_, the intensity of PL signal for the rGO/TiO_2_ and NS-rGO/TiO_2_ nanocomposites was much lower. This indicated the photogenerated electrons from TiO_2_ was transferred into carbon atoms on the graphene, then the charge recombination was reduced, while NS-rGO was more efficient for surface oxygen vacancies and defects. This further verified that the NS-rGO decorated TiO_2_ was more beneficial than rGO for the electron/hole separation.

TiO_2_ and rGO (or NS-rGO) were mixed with a mass ratio of 100:1 at room temperature, resulting in rGO/TiO_2_ and NS-rGO/TiO_2_, respectively. The concentration of graphene in the prepared rGO/TiO_2_ and NS-rGO/TiO_2_ was also characterized by TGA (Figure S7). The total weight loss of rGO/TiO2 (4.67%) or NS-rGO/TiO_2_ (0.92%) could be ascribed to the loss of the adsorbed H_2_O, the crystal transfer of TiO_2_ and the oxidation of rGO or NS-rGO. From the TGA results, the real content of rGO or NS-rGO in the nanocomposite could not be estimated , but low concentration of graphene was confirmed which was consistent with the dosage of theory (100:1 of TiO_2_ : rGO/NS-rGO). The BET was also added, and the high surface area and porous structure might be the reason for the high activity of NS-rGO/TiO_2_. Because the BET surface area of rGO/TiO_2_ and NS-rGO/TiO_2_ was 53.2 m^2^/g and 54.2 m^2^/g, respectively, and their pore distribution was also similar (Figure S8), the comparison between NS-rGO/TiO_2_ and rGO/TiO_2_ could be used to disclose the influence of graphene structure on the photocatalytic H_2_ production activity.

**Figure 8:**
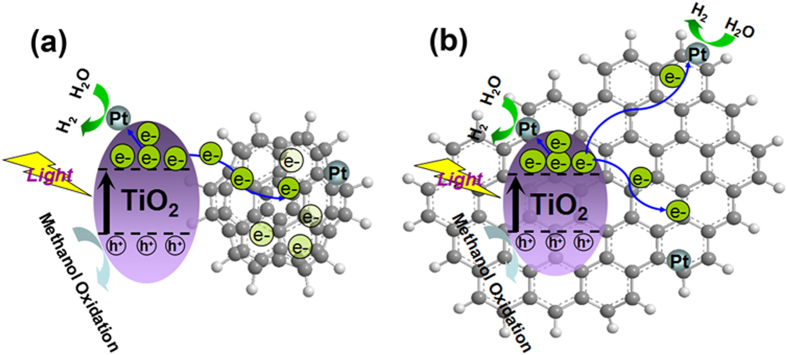
Illustration of the working principle of (**a**) NS-rGO/TiO_2_ (**b**) rGO/TiO_2_ and photocatalytic system for hydrogen production under UV-Vis irradiation.

On the basis of the above results, a tentative mechanism of the photocatalytic reaction was proposed as illustrated in Fig. 8. When irradiated under visible light, the electron/hole (e^−^/h^+^) pairs from TiO_2_ were excited and formed. The electrons were most likely to transfer in one of three following ways to: (1) Pt deposited on the surface of TiO_2_ nanoparticles; (2) carbon atoms on the graphene; or (3) Pt located on the graphene^7^. Eventually, the electrons were reacted with the adsorbed H^+^ ions for H_2_ production. This mechanism almost faultlessly explained why rGO/TiO_2_ and NS-rGO/TiO_2_ could significantly enhance photocatalytic activity. With the increasing of irradiation time from 1 h–5 h, the photocatalytic H_2_ production rate of NS-rGO/TiO_2_ was three times higher than rGO/TiO_2_. In this regard, when electrons were transferred to carbon atoms on the graphene, the flat structure of rGO could help electrons freely travel all around and then the opportunity for electrons/holes recombination was enough (Fig. 8a). While the sphere structure of NS-rGO contained multilayer graphene sheets was used, electrons not only could freely travel on the graphene sheets like as rGO, but also transfer from outer graphene sheets to inner sheets. It might be noticed that the inner electrons were tentatively stored and almost no chance to be reacted. This could be explained as electrons warehouse in the NS-rGO (Fig. 8b). Once NS-rGO was introduced to the TiO_2_ to form hybrid catalyst, it could efficiently suppress charge recombination, improve interfacial charge transfer, furthermore, the unique feature of NS-rGO could provide more active adsorption sites, photocatalytic reaction centers and reaction space than that of rGO. As a consequence, a suitable structure of graphene was crucial for providing the photocatalytic activity and stability of graphene based photocatalysts.

## Methods

### Materials

Graphite powder was purchased from national medicine group chemical reagent Co., Ltd. in China. Sodium nitrate, potassium permanganate, hydrochloric acid, sulfuric acid, barium chloride, and hydrogen peroxide (30%) were analytical grade and purchased from Shantou west long chemical Co., Ltd in China. Iron (III) chloride anhydrous (98%) was purchased from Alfa Aesar. Nano-TiO_2_ nanoparticles were purchased from Alfa Aesar. All chemicals and solvents were used as received. All aqueous solutions were prepared using ultrapure water (18 MU) from a Milli-Q system (Millipore).

### Instrumentation

Transmission electron microscopy (TEM) was conducted by a Tecnai G2 20 S-TWIN.TEM instrument was operated at 200 kV. Scanning electron microscope (SEM) imaging and EDX chemical analysis were conducted using a JSM-6010La scanning electron microscope. Using Cu-Kα radiation (λ = 0.1541 nm), the X-ray diffraction (XRD) patterns of all samples were recorded with a D/MAX in Japan-TTRIII diffractometer and the data were collected from 10° to 80° (2 Theta). UV-vis diffuse reflectance spectra were measured on a Hitachi spectrophotometer using BaSO_4_ as a reference. Band gap energies were calculated by analysis of the Tauc-plots resulting from Kubelka-Munk transformation of diffuse reflectance spectra, which can be estimated from the intersection point of the linear part of the plot (*F(R)·hv*)^1/2^ versus *hv* with the photon energy axis. The fluorescence (FL) measurements were performed on a Hitachi U-3010 spectrophotometer at room temperature.The excitation wavelength was 315 nm. Raman spectrum was performed by using a laser raman spectrometer (LabRAM HR800) at room temperature. The Brunauer-Emmett-Teller (BET) method was utilized to calculate the specific surface areas (S_BET_). Thermogravimetric analysis (TGA) was performed using a NETZSCH TG 209 F1 thermogravimetric analyzer from room temperature to 800 ^o^C with a heating rate of 10 ^o^C min^−1^ and an N_2_ flow rate of 20 mL min^−1^.

### Preparation of graphene oxide

Graphene oxide (GO) was prepared according to a modified Hummer method^32^. Typically, graphite powder (1.0 g) was mixed with concentrated sulfuric acid (23 mL) in a 1 L round bottom flask and stirred in an ice bath. NaNO_3_ (0.5 g) and KMnO_4_ (3.0 g) were slowly added into the suspension and kept the temperature at 0 ± 1 °C. After keeping the mixture at 35 ± 3 °C for 30 min, water (46 mL) was slowly added, the suspension was heated up to 98 °C and maintained for 15 min, and then 140 mL of water and 2 mL of H_2_O_2_ (30%) were added to end the reaction. Thereafter, the suspension was hot filtered, washed by HCl solution (5%) until no SO_4_^2−^ could be detected in the filtrate, the products were vacuum-dried at 60 °C over-night and then sealed for the preservation.

### Preparation of NS-rGO and rGO

NS-rGO was prepared by a simple two-step reaction. Reduced graphene oxide coated α-Fe_2_O_3_ nanopellets were prepared as follows. Ferric chloride (1.0 g) and anhydrous sodium acetate (1.0 g) were dissolved in diethylene glycol (20 mL) and then graphene oxide solution (5.0 mg mL^−1^, 5 mL) was added. The mixture was dispersed by ultrasound for 30 min, transferred into a teflonlined stainless-steel autoclave, heated to and maintained at 200 °C for 10 h, and then cooled to room temperature. The black products were washed several times with ethanol and water. The brownish red solid was obtained and dried at 80 °C. Hydrochloric acid (6 mol L^−1^, 30 mL) was added into above mentioned solid, keeping for one day. After that, the mixture was filtered with 0.22 μm filter membrane, and washed by ultrabare water until the filtrate was neutral. The black and feathery solid was dried at 80 °C and then NS-rGO was obtained. rGO was prepared similar to NS-rGO except that no Ferric chloride was added.

### Preparation of photocatalyst (rGO/TiO_2_, NS-rGO/TiO_2_, Pt-TiO_2_, Pt-rGO/TiO_2_ and Pt-NS-rGO/TiO_2_)

The hybrid photocatalysts were synthesized by directly mixing. TiO_2_ (0.5 g) was dissolved in 20 mL of ethanol with stirring. Then, rGO or NS-rGO (5 mg) was mixed with TiO_2_-ethanol solution by ultrasonic for 1 h at room temperature. After that the samples were dried in an air oven at 80 °C for 24 h and then rGO/TiO_2_ and NS-rGO/TiO_2_ nanocomposites were obtained, respectively. In order to synthesize Pt-rGO/TiO_2_ and Pt-NS-rGO/TiO_2_, the photocatalytic deposition method was carried out in the presence of nanocomposites (rGO/TiO_2_ or NS-rGO/TiO_2_) and chloroplatinic acid (H_2_PtCl_6_) in methanol solution (1 mol L^−1^). The platinum deposition was achieved under UV irradiation for 30 min using a 200-W mercury lamp. The amount of platinum was fixed at 0.1 wt % among TiO_2_, rGO/TiO_2_, or NS-rGO/TiO_2_.

### Photocatalytic reaction

Water splitting reactions were carried out in a Pyrex vessel connected to a glass closed circulation and evacuation system. The light source was a 300 W Xe arc lamp (Perfectlight Co., PLS-SXE300). Prior to irradiation, the whole system, including the photocatalysts was purged with argon at 100 N mL/min to remove the air completely. High-purity Argon (99.9999%) was used as a carrier gas for the reaction products of which continuous gas flow was set to 50 N mL min^−1^. Gas chromatography equipped with a thermal conductivity detector and molecular sieve 5-Å column with Ar carrier gas was used for the determination of hydrogen concentration. In a typical run, 50 mg of photocatalyst powder was suspended in an 80 mL, 20% v/v methanol aqueous solution. Here, platinum and methanol were added as co-catalyst and sacrificial reagent, respectively. The apparent quantum efficiency (QE) was measured under the same photocatalytic reaction condition with four 365 nm-LEDs used as light sources to trigger the photocatalytic reaction, and the QE was calculated according to equation (1)


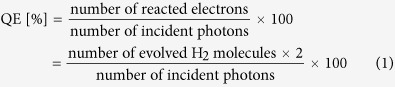


## Additional Information

**How to cite this article**: Chen, D. *et al*. Nanospherical like reduced graphene oxide decorated TiO_2_ nanoparticles: an advanced catalyst for the hydrogen evolution reaction. *Sci. Rep*. **6**, 20335; doi: 10.1038/srep20335 (2016).

## Supplementary Material

Supplementary Information

## References

[b1] Paracchino A. . Highly active oxide photocathode for photoelectrochemical water reduction. Nat. Mater. 10, 456–461 (2011). 21552270 10.1038/nmat3017

[b2] Fujishima A. . Electrochemical photolysis of water at a semiconductor electrode. Nature 238, 37–38 (1972). 12635268 10.1038/238037a0

[b3] Li S. . Water-soluble and lowly toxic sulphur quantum dots. Adv. Funct. Mater. 45, 7133–7138 (2014).

[b4] Zuo F. . Active facets on titanium (III)-doped TiO_2_: an effective strategy to improve the visible-light photocatalytic activity. Angew. Chem. Int. Ed. 124, 6327–6330 (2012). 10.1002/anie.201202191 22566101

[b5] Lu X. . Efficient photocatalytic hydrogen evolution over hydrogenated ZnO nanorod arrays. Chem. Commun. 48, 7717–7719 (2012). 10.1039/c2cc31773g 22544331

[b6] Gao P. . The synergetic effect of sulfonated graphene and silver as co-catalysts for highly efficient photocatalytic hydrogen production of ZnO nanorods. J. Mater. Chem. A 1, 14262–14269 (2013).

[b7] Li Q. . Highly efficient visible-light-driven photocatalytic hydrogen production of CdS-cluster-decorated graphene nanosheets. J. Am. Chem. Soc. 133, 10878–10884 (2011). 21639097 10.1021/ja2025454

[b8] Bao N. . Self-templated synthesis of nanoporous CdS nanostructures for highly efficient photocatalytic hydrogen production under visible light. Chem. Mater. 20, 110–117 (2007).

[b9] Li Y. . Synthesis of CdS nanorods by an ethylenediamine assisted hydrothermal method for photocatalytic hydrogen evolution. J. Phys. Chem. C 113, 9352–9358 (2009).

[b10] Zhang J. . Nanospherical carbon nitride frameworks with sharp edges accelerating charge collection and separation at a soft photocatalytic interface. Adv. Mater. 26, 4121–4126 (2014). 24706532 10.1002/adma.201400573

[b11] Martin D. . Highly efficient photocatalytic H_2_ evolution from water using visible light and structure-controlled graphitic carbon nitride. Angew. Chem. Int. Ed. 53, 9240–9245 (2014). 10.1002/anie.201403375 PMC4257501 25045013

[b12] Wang X. . A metal-free polymeric photocatalyst for hydrogen production from water under visible light. Nat. Mater. 8, 76–80 (2008). 18997776 10.1038/nmat2317

[b13] Higashi M. . Photocatalytic overall water splitting under visible light using ATaO_2_N (A = Ca, Sr, Ba) and WO_3_ in a IO_3_^−^/I^−^ shuttle redox mediated system. Chem. Mater. 21, 1543–1549 (2009).

[b14] Jia Q. . Facile fabrication of an efficient BiVO_4_ thin film electrode for water splitting under visible light irradiation. P. Natl. Acad. Sci. USA 109, 11564–11569 (2012). 10.1073/pnas.1204623109 PMC3406872 22699499

[b15] Kamegawa T. . A visible-light-harvesting assembly with a sulfocalixarene linker between dyes and a Pt-TiO_2_ photocatalyst. Angew. Chem. Int. Ed. 52, 916–919 (2013). 10.1002/anie.201206839 23197327

[b16] Chen X. . Semiconductor-based photocatalytic hydrogen generation. Chem. Rev. 110, 6503–6570 (2010). 21062099 10.1021/cr1001645

[b17] Eda G. . Chemically derived graphene oxide: towards large-area thin-film electronics and optoelectronics. Adv. Mater. 22, 2392–2415 (2010). 20432408 10.1002/adma.200903689

[b18] Lee C. . Measurement of the elastic properties and intrinsic strength of monolayer graphene. Science 321, 385–388 (2008). 18635798 10.1126/science.1157996

[b19] Balandin A. . Superior thermal conductivity of single-layer graphene. Nano Lett. 8, 902–907 (2008). 18284217 10.1021/nl0731872

[b20] Zhu Y. . Graphene and graphene oxide: synthesis, properties, and applications. Adv. Mater. 22, 3906–3924 (2010). 20706983 10.1002/adma.201001068

[b21] Xiang Q. . Synergetic effect of MoS_2_ and graphene as cocatalysts for enhanced photocatalytic H_2_ production activity of TiO_2_ nanoparticles. J. Am. Chem. Soc. 134, 6575–6578 (2012). 22458309 10.1021/ja302846n

[b22] Fan W. . Nanocomposites of TiO_2_ and reduced graphene oxide as efficient photocatalysts for hydrogen evolution. J. Phys. Chem. C 115, 10694–10701 (2011).

[b23] Xiang Q. . Graphene-based semiconductor photocatalysts. Chem. Soc. Rev. 41, 782–796 (2012). 21853184 10.1039/c1cs15172j

[b24] Zhang X. . Graphene/TiO_2_ nanocomposites: synthesis, characterization and application in hydrogen evolution from water photocatalytic splitting. J. Mater. Chem. 20, 2801–2806 (2010).

[b25] Iwase A. . Reduced graphene oxide as a solid-state electron mediator in Z-scheme photocatalytic water splitting under visible light. J. Am. Chem. Soc. 133, 11054–11057 (2011). 21711031 10.1021/ja203296z

[b26] Wang Y. . Electrostatic self-assembly of BiVO_4_-reduced graphene oxide nanocomposites for highly efficient visible light photocatalytic activities. ACS Appl. Mater. Inter. 6, 12698–12706 (2014). 10.1021/am502700p 25010256

[b27] Xiang Q. . Enhanced photocatalytic H_2_-production activity of graphene-modified titania nanosheets. Nanoscale 3, 3670–3678 (2011). 21826308 10.1039/c1nr10610d

[b28] Fan W. . Fabrication of TiO_2_/RGO/Cu_2_O heterostructure for photoelectrochemical hydrogen production. Appl. Catal. B-Environ. 181, 7–15 (2016).

[b29] Moon G. H. . Solar production of H_2_O_2_ on reduced graphene oxide-TiO_2_ hybrid photocatalysts consisting of earth-abundant elements only. Energy Environ. Sci. 7, 4023–4028 (2014).

[b30] Mou Z. . TiO_2_ Nanoparticles-functionalized N-doped graphene with superior interfacial contact and enhanced charge separation for photocatalytic hydrogen generation. ACS Appl. Mater. Inter. 6, 13798–13806 (2014). 10.1021/am503244w 25078680

[b31] Moon G. . Platinum-like behavior of reduced graphene oxide as a cocatalyst on TiO_2_ for the efficient photocatalytic oxidation of arsenite. Environ. Sci. Technol. Lett. 1, 185–190 (2014).

[b32] Wang C. . Enhanced photo-electrocatalytic performance of Pt/RGO/TiO_2_ on carbon fiber towards methanol oxidation in alkaline media. J. Solid State Electr. 18, 515–522 (2014).

[b33] Xiang Q. . Graphene-based photocatalysts for solar fuel generation, Angew. Chem. Int. Ed. 54, 11350–11366 (2015). 10.1002/anie.201411096 26079429

[b34] Xiang Q. . Graphene-based photocatalysts for hydrogen generation, J. Phys. Chem. Lett. 4, 753–759 (2013). 26281930 10.1021/jz302048d

[b35] Liu L. . Engineering the TiO_2_-graphene interface to enhance photocatalytic H_2_ production. ChemSusChem. 7, 618–626 (2014). 24323576 10.1002/cssc.201300941

[b36] Xing M. . Synergistic effect on the visible light activity of Ti^3+^ doped TiO_2_ nanorods/boron doped graphene composite. Sci. Rep. 4 (2014). 10.1038/srep05493 PMC4074785 24974890

[b37] Gu Y. . Synthesis and photocatalytic activity of graphene based doped TiO_2_ nanocomposites, Appl. Surf. Sci. 319, 8–15 (2014).

[b38] Li H. . A hydrothermal route for constructing reduced graphene oxide/TiO_2_ nanocomposites: Enhanced photocatalytic activity for hydrogen evolution, Int. J. Hydrogen Energy 39, 19877–19886 (2014).

[b39] Nai J. . Structure-dependent electrocatalysis of Ni[OH]_2_ hourglass-like nanostructures towards L-histidine. Chem. -Eur. J. 19, 501–508 (2013). 23255517 10.1002/chem.201203009

[b40] Xu Y. . Facile fabrication of reduced graphene oxide encapsulated copper spherical particles with 3D architecture and high oxidation resistance. RSC Adv. 4, 58005–58010 (2014).

[b41] Chen D. . Unique lead adsorption behavior of ions sieves in pellet-like reduced graphene oxide. RSC Adv. 5, 73333–73339 (2015).

[b42] Mahmoudian M. . Synthesis and characterization of Fe_3_O_4_ rose like and spherical/reduced graphene oxide nanosheet composites for lead (II) sensor. Electrochim. Acta 169, 126–133 (2015).

[b43] Li S. . Yolk-shell hybrid nanoparticles with magnetic and pH-sensitive properties for controlled anticancer drug delivery. Nanoscale 5, 11718–11724 (2013). 24107975 10.1039/c3nr04032a

[b44] Chai B. . Synthesis of C_60_-decorated SWCNTs (C_60_-d-CNTs) and its TiO_2_-based nanocomposite with enhanced photocatalytic activity for hydrogen production. Dalton Trans. 42, 3402–3409 (2013). 23258545 10.1039/c2dt32458j

[b45] Li H. . Water-soluble fluorescent carbon quantum dots and photocatalyst design. Angew. Chem. Int. Ed. 49, 4430–4434 (2010). 10.1002/anie.200906154 20461744

[b46] Chen W. . Record high hole mobility in polymer semiconductors via side-chain engineering. J. Am. Chem. Soc. 133, 14896–14899 (2011). 24053786 10.1021/ja405112s

[b47] Tang J. . Photophysical and photocatalytic properties of AgInW_2_O_8_. J. Phys. Chem. B 107, 14265–14269 (2003).

[b48] Eda G. . Blue photoluminescence from chemically derived graphene oxide. Adv. Mater. 22, 505–509 (2010). 20217743 10.1002/adma.200901996

