# Supplementary material for: Nanospherical like reduced graphene oxide decorated TiO2 nanoparticles: an advanced catalyst for the hydrogen evolution reaction
Source: Sci Rep. 2016 Feb 1;6:20335. doi: 10.1038/srep20335 (PMC4734335; doi:10.1038/srep20335)
Supplement: Supplementary Information [file srep20335-s1.doc]

**Supporting Information**

**Nanospherical like reduced graphene oxide decorated TiO_2_ nanoparticles: an advanced catalyst for the hydrogen evolution reaction**

Dejian Chen, Liling Zou, Shunxing Li* & Fengying Zheng

*College of Chemistry and Environment, Minnan Normal University, 36 Xianqian Street, Zhangzhou 363000*

*Correspondence should be addressed to Shunxing Li (email: lishunxing@mnnu.edu.cn or shunxing_li@aliyun.com)*

**
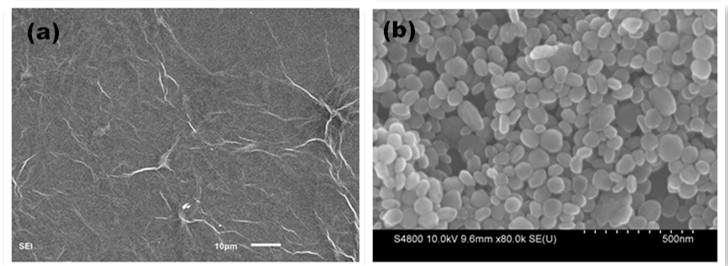
**

**Figure S1.** (a) SEM image of graphene oxide. (b) Typical SEM image of α-Fe_2_O_3_@rGO nanoparticles.

SEM characterization was used for illuminating the size and morphology of pure graphene oxide and shown in Figure S 1a. It was clearly indicated that the morphology of graphene oxide was lamellar structure. The size of graphene oxide at the micron-level. The SEM image (Figure S 1b) clearly confirmed the morphology of synthesized α-Fe_2_O_3_@rGO nanoparticles are monodispersion, nanosize, and uniform elliptical structure. No large layers of graphene were observed.


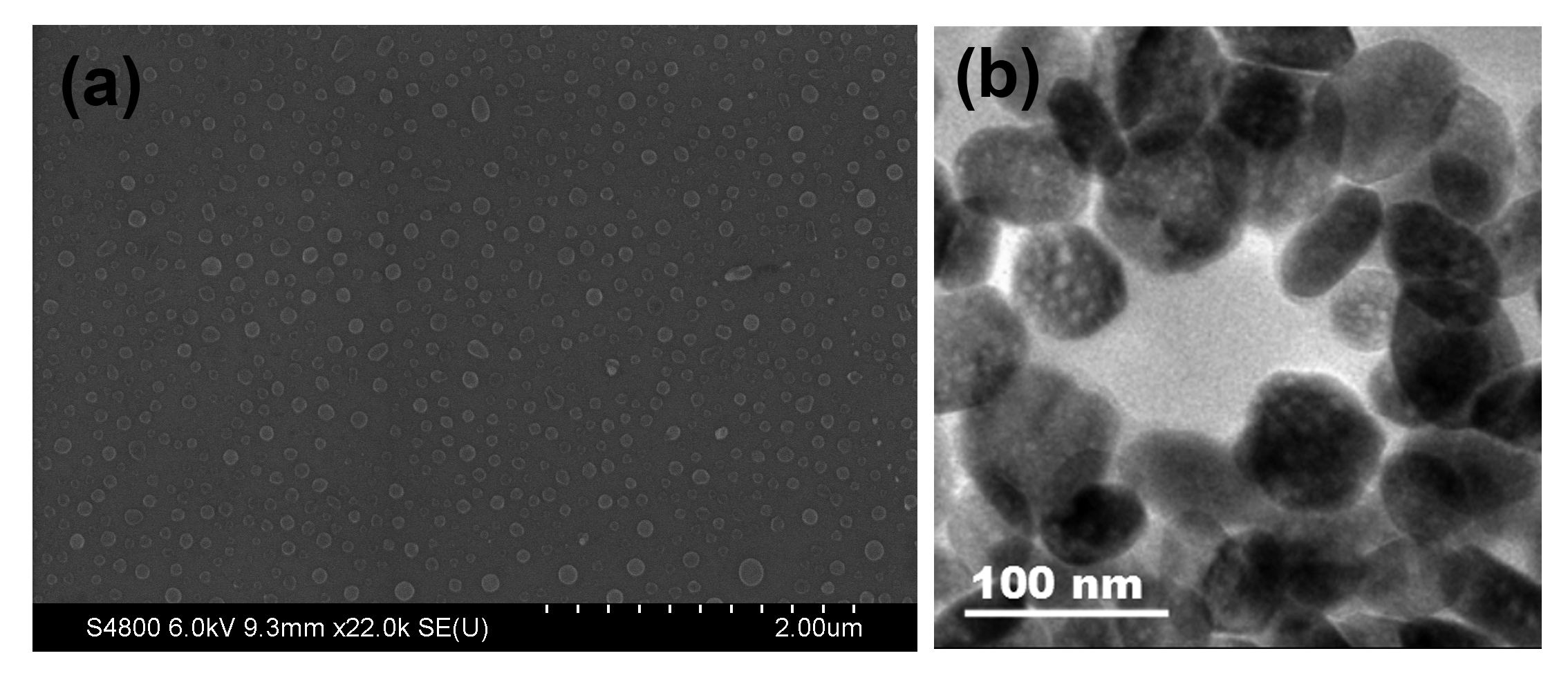


**Figure S2.** (a) SEM and (b) TEM image of α-Fe_2_O_3_@rGO nanoparticles.

The SEM and TEM image (Figure S 2) clearly confirmed the synthesized α-Fe_2_O_3_@rGO nanoparticles were monodispersion, nanosize, and uniform elliptical morphology.

**
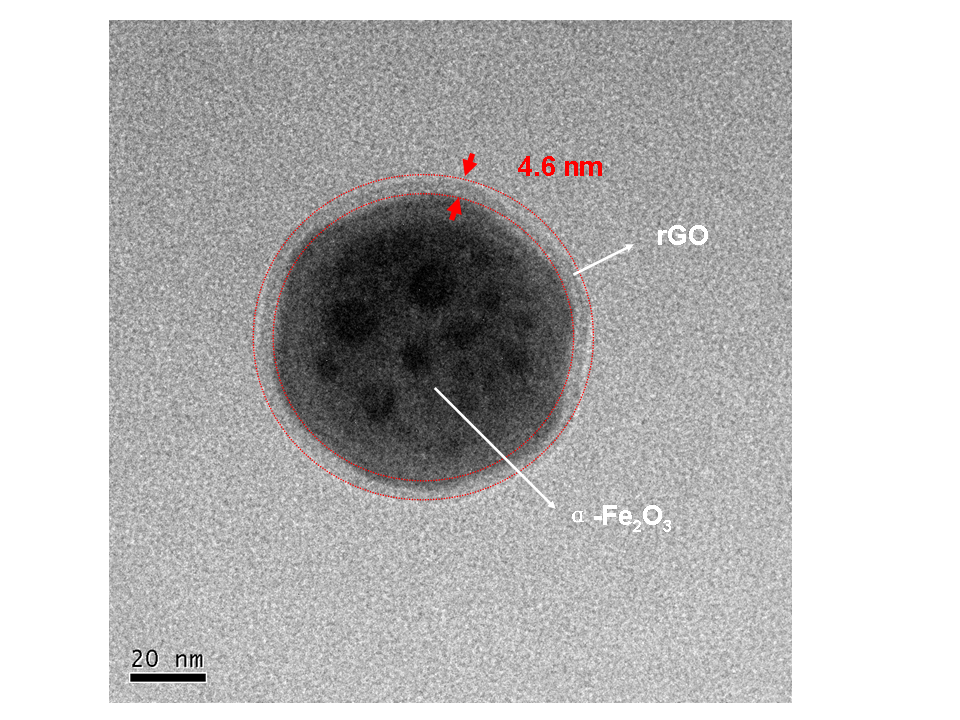
**

**Figure S3.** Typical TEM image of a single α-Fe_2_O_3_@rGO nanoparticles.

To further observe the morphology and structure of the composites, TEM was used to characterize the three dimensional structure of *α*-Fe_2_O_3_@rGO nanoparticles. As shown in Figure S3, the composites exhibited a notable core-shell structure and their core and shell were *α*-Fe_2_O_3_ and rGO, respectively. The rGO sheets could be uniformly coated onto *α*-Fe_2_O_3_ particles, yielding elliptical morphology. The thickness of outer rGO layer was about 4.6 nm.





**Figure S4.** TEM image of NS-rGO.

NS-rGO was characterized by TEM. NS-rGO were sphere like particles with lofted surface, because they were prepared by acidic etching.

**
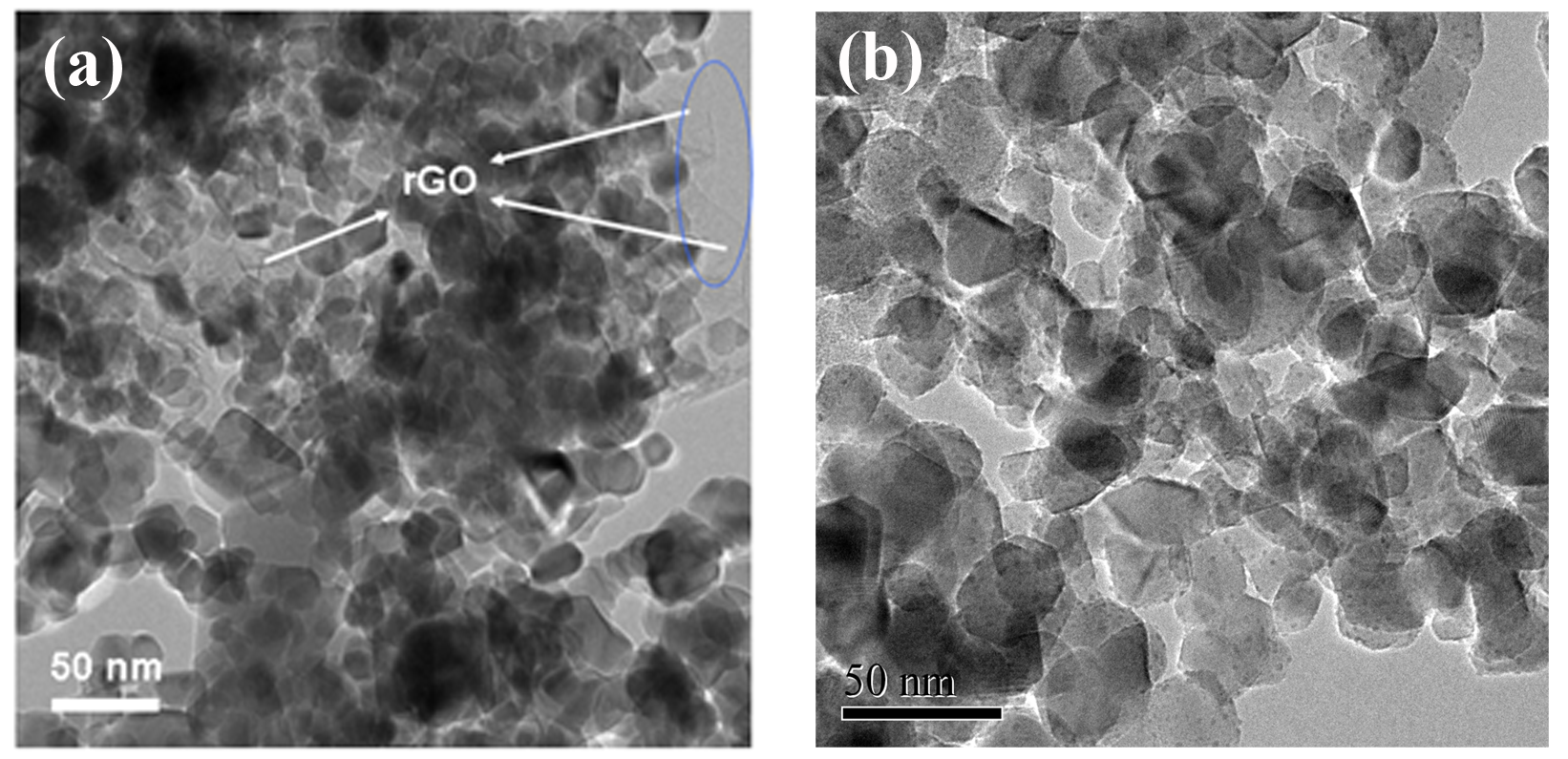
**

**Figure S5** (a) Typical TEM image of rGO/TiO_2_ nanocompositions. (b) Typical TEM image of NS-rGO/TiO_2_ deposited with Pt nanoparticles.

According to a mass ratio of 100:1, TiO_2_ and rGO were mixed at room temperature to form gray suspension. Transmission electronic microscope (TEM) was employed to observe its morphology. Figure S 5a clearly showed that the nanocomposites of rGO sheet and TiO_2_ nanoparticles, the sheet structure of rGO was observed in blue line area, indicated forming composite structure among them. Figure S 5b clearly showed that the Pt nanoparticles were uniform deposited on the surface of NS-rGO/TiO_2_ nanocompositions.


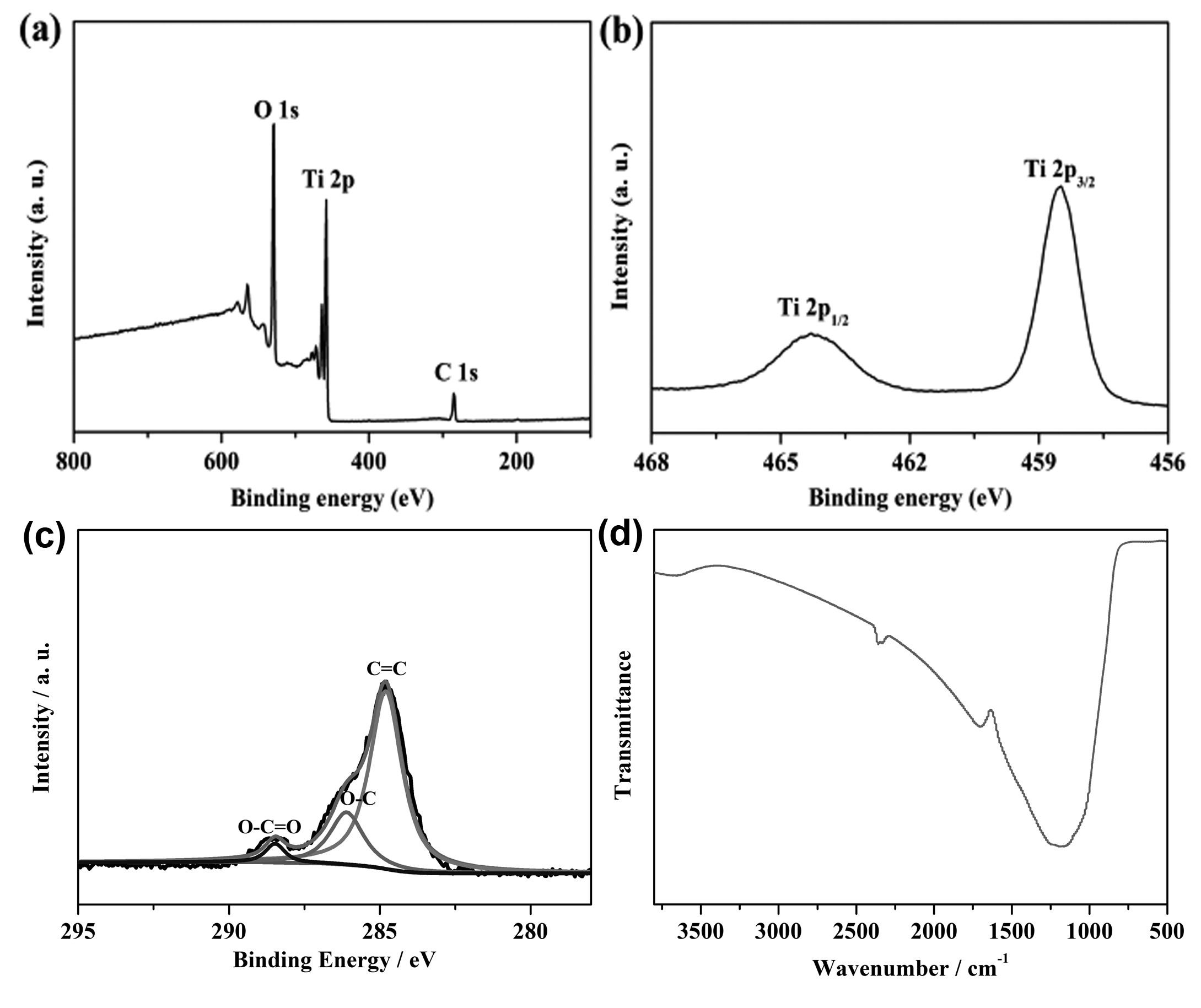


**Figure S6.** (a) The XPS survey spectrum, (b) Ti 2p, (c) C 1s, and (d) FTIR spectrum of NS-rGO/TiO_2_ nanocompositions.

The XPS survey spectra of NS-rGO/TiO_2_ within the binding energy range from 100 to 800 eV indicated that the existence of NS-rGO in sample. As shown in Figrure 6b, the core levels of Ti 2p_1/2_ and Ti 2p_3/2_ were approximately at 464.3 eV and 458.5 eV, respectively, which were assigned to the Ti^4+^ in TiO_2_. .C 1s XPS characterizations were added in Figure S6c, the results indicated that there was not Ti-O-C generation between TiO_2_ and NS-rGO. The C 1s spectrum of the NS-rGO/TiO_2_ showed one strong peak at 284.8 eV, two weak peaks at 286.1 and 288.2 eV, which were attributed to the elemental carbon and the carbonate species, respectively. The peak ascribed to Ti-C bonds at around 281 eV was not detected in the NS-rGO/TiO_2_, suggesting that carbon was not doped into the lattice of TiO_2_. From the IR spectrum (Figure 6d), any peaks attributed Ti-O-C bands were not observed.





**Figure S7.** TGA curves of rGO/TiO_2_ and NS-rGO/TiO_2_.

TiO_2_ and rGO (or NS-rGO) were mixed with a mass ratio of 100:1 at room temperature, resulting in rGO/TiO_2_ and NS-rGO/TiO_2_, respectively. The concentration of graphene in the prepared rGO/TiO_2_ and NS-rGO/TiO_2_ was also characterized by TGA (Figure S7). The total weight loss of rGO/TiO_2_ (4.67 %) or NS-rGO/TiO_2_ (0.92 %) could be ascribed to the loss of the adsorbed H_2_O, the crystal transfer of TiO_2_ and the oxidation of rGO or NS-rGO.





**Figure S8.** Nitrogen adsorption-desorption isotherms of rGO/TiO_2_ and NS-rGO/TiO_2_. Inset is pore distribution curve.

The BET surface area of the rGO/TiO_2_ and NS-rGO/TiO_2_ was 53.2 m^2^/g and 54.2 m^2^/g, respectively. The increased specific surface area and porous was attributed to the hollow and spherical like structure of NS-RGO.
